# A paradoxical increase of force development in saphenous and tail arteries from heterozygous ANO1 knockout mice

**DOI:** 10.14814/phy2.14645

**Published:** 2020-11-27

**Authors:** Vladimir V. Matchkov, Henrik Black Joergensen, Dmitrii Kamaev, Andreas Hoegh Jensen, Hans Christian Beck, Boris V. Skryabin, Christian Aalkjaer

**Affiliations:** ^1^ Department of Biomedicine, MEMBRANES, Health Aarhus University Aarhus Denmark; ^2^ Department for Clinical Biochemistry and Pharmacology University of Southern Denmark Odense Denmark; ^3^ Medical Faculty Core Facility Transgenic Animal and Genetic Engineering Models (TRAM) University of Muenster Muenster Germany

**Keywords:** ANO1, calcium‐activated chloride channel, intracellular calcium, small artery contraction, smooth muscle

## Abstract

A Ca^2+^‐activated Cl^−^ channel protein, ANO1, is expressed in vascular smooth muscle cells where Cl^−^ current is thought to potentiate contraction by contributing to membrane depolarization. However, there is an inconsistency between previous knockout and knockdown studies on ANO1’s role in small arteries. In this study, we assessed cardiovascular function of heterozygous mice with global deletion of exon 7 in the ANO1 gene. We found decreased expression of ANO1 in aorta, saphenous and tail arteries from heterozygous ANO1 knockout mice in comparison with wild type. Accordingly, ANO1 knockdown reduced the Ca^2+^‐activated Cl^−^ current in smooth muscle cells. Consistent with conventional hypothesis, the contractility of aorta from ANO1 heterozygous mice was reduced. Surprisingly, we found an enhanced contractility of tail and saphenous arteries from ANO1 heterozygous mice when stimulated with noradrenaline, vasopressin, and K^+^‐induced depolarization. This difference was endothelium‐independent. The increased contractility of ANO1 downregulated small arteries was due to increased Ca^2+^ influx. The expression of L‐type Ca^2+^ channels was not affected but expression of the plasma membrane Ca^2+^ ATPase 1 and the Piezo1 channel was increased. Expressional analysis of tail arteries further suggested changes of ANO1 knockdown smooth muscle cells toward a pro‐contractile phenotype. We did not find any difference between genotypes in blood pressure, heart rate, pressor response, and vasorelaxation in vivo. Our findings in tail and saphenous arteries contrast with the conventional hypothesis and suggest additional roles for ANO1 as a multifunctional protein in the vascular wall that regulates Ca^2+^ homeostasis and smooth muscle cell phenotype.

## Graphical abstract

ANO1 is not only the chloride channel but also a regulator of smooth muscle cell contractile phenotype.

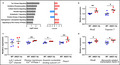

## 1 INTRODUCTION

ANO1 protein, also known as TMEM16a, is a Ca^2+^‐activated Cl^−^ channel (Caputo et al., 2008; Schroeder et al., 2008; Yang et al., 2008) that is known to be expressed in different tissues (Pedemonte & Galietta, 2014). In particular, ANO1 was shown to be expressed in vascular smooth muscle cells (Dam et al., 2014; Davis et al., 2010; Manoury et al., 2010; Thomas‐Gatewood et al., 2011) where Ca^2+^‐activated Cl^−^ channels are thought to potentiate contraction by contributing to smooth muscle cell depolarization (Criddle et al., 1996; Large & Wang, 1996). Activation of Ca^2+^‐activated Cl^−^ channels may be initiated by release of Ca^2+^ from intracellular stores (Yang et al., 2008) or Ca^2+^ influx through transient receptor potential channels (Wang et al., 2016) and lead to efflux of negatively charged Cl^−^ ions from the cell causing depolarization and activation of voltage‐sensitive L‐type Ca^2+^ channels (Dam et al., 2014). Influx of Ca^2+^ through L‐type Ca^2+^ channels leads to an increase in the global intracellular Ca^2+^ concentration and activation of the contractile machinery of smooth muscle cells.

This potentially makes ANO1 an important protein involved in the regulation of the tone of small arteries and hence a regulator of vascular resistance, which defines blood flow and arterial pressure (Mendelsohn, 2005; Mulvany & Aalkjaer, 1990). Consistent with this role, ANO1 has been found to be upregulated in experimental models with circulatory abnormalities, for example, pulmonary and essential hypertension (Askew Page et al., 2019; Forrest et al., 2012; Gui et al., 2015; Papp et al., 2019; Wang et al., 2015). Investigation of the functional importance of Cl^−^ channels in blood vessels is however, difficult due to the absence of specific pharmacological tools (Boedtkjer et al., 2015; Greenwood & Leblanc, 2007). Alternatively, knockout and knockdown animal models are used to provide information about protein function in vitro and in vivo (Bulley et al., 2012; Dam, et al., 2014; Heinze et al., 2014; Jensen et al., 2018).

Homozygous global knockout of ANO1 in mice was demonstrated to be lethal (Heinze et al., 2014). We previously studied segments of rat mesenteric small arteries with siRNA‐induced knockdown of ANO1 and found the expected reduction in arterial contractility (Dam, et al., 2014). Knockdown of ANO1 in mouse cerebral small arteries also leads to reduced arterial contractility (Bulley et al., 2012). Heinze et al. (2014) developed a mouse with smooth muscle‐specific deletion of exon 21 of ANO1. In these mice, the contractility of aorta was reduced, while the contractility of small mesenteric arteries, which had little ANO1‐mediated current, from knockout and wild type mice was similar. This is consistent with the previous report on mice constitutively expressing siRNA against ANO1 in their smooth muscle cells where no changes in the contractility of small mesenteric and femoral arteries was seen (Jensen et al., 2018). However, this previous study reported also that tail artery had reduced contractility to agonist stimulation when ANO1 was downregulated by constitutive expression of ANO1‐siRNA. Surprisingly, this ANO1 downregulation also suppressed contraction to K^+^‐induced depolarization that cannot be explained by the conventional model for the role of ANO1 in membrane potential dependent potentiation of agonist‐induced contraction (Jensen et al., 2018). This effect of ANO1 downregulation was explained by reduced expression of the L‐type Ca^2+^ channels and hence voltage‐gated Ca^2+^ influx. Potential problems in these models come from possible off‐target effect of siRNA in the knockdown models (Bulley et al., 2012; Dam, et al., 2014; Jensen et al., 2018) and the fact that in the smooth muscle‐specific deletion model the Ca^2+^‐activated Cl^−^ currents were suppressed while ANO1 protein expression (albeit at a lower molecular weight) was still present (Heinze et al., 2014).

To get a comprehensive understanding of ANO1’s role in the vasculature, it is relevant to study different genetic models. In this paper, we describe the cardiovascular function of mice with heterozygous global knockout of ANO1. We aimed to test whether genetic knockdown of the ANO1 protein can reproduce the siRNA‐induced downregulation studies (Bulley et al., 2012; Dam, et al., 2014; Jensen et al., 2018) and support results from smooth muscle‐specific deletion of ANO1 in mice (Heinze et al., 2014) in terms of blood pressure control and small artery function. We deleted another exon (exon 7) of ANO1 than the previously published exon 21 (Heinze et al., 2014), which resulted in deletion of ANO1 protein expression. In these mice, ANO1 expression in aorta and in saphenous and tail arteries was reduced. Consistently, the Ca^2+^‐activated Cl^−^ current was reduced in ANO1 downregulated tail artery smooth muscle cells. Consistent with previous reports and with theoretical considerations the contractility of aorta from the heterozygous mice was reduced compared with aorta from wild type mice. Surprisingly, we found an enhanced elevation of intracellular Ca^2+^ and contractility of tail and saphenous arteries from heterozygous mice when activated with noradrenaline and vasopressin. This finding contrasts with the hypothesis that ANO1 acts as an amplifier of agonist‐induced contraction in all vascular bed and suggests additional roles of ANO1. Our findings suggest a role for increased Ca^2+^ influx, possibly because of changes in the expression of Ca^2+^ influx pathways in smooth muscle cells. Despite the increased contractility of some arteries from the heterozygous mice, we did not detect any difference in blood pressure between ANO1 knockdown and wild type mice.

## 2 METHODS

### 2.1 Targeting construct design

**FIGURE 1:**
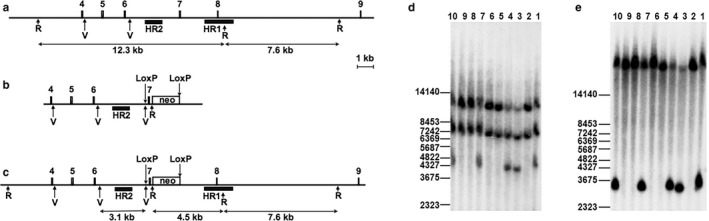
Targeting of the exon 7 of mouse ANO1 gene. (a–c) The intron and intergenic regions are shown as lines, exons are shown as filled boxes. The empty box corresponds to the neomycin resistance cassette (neo) flanked by the FRT sites (not shown). Exons numeration is shown above. The arrows above correspond to the LoxP sequences, and arrows below corresponding to restriction endonuclease sites *Eco*RI (R) and *Eco*RV (V). The black boxes correspond to Southern probes sequences (HR1 and HR2 as indicated). The expected sizes of restriction DNA fragments are labeled below in kb. (a) Wild type locus. (b) Targeted vector structure without negative selection marker and plasmid backbone. (c) Genomic locus after the homologous recombination. The neomycin cassette is present in intron 7, and exon 7 flanked by two LoxP sites. (d) Southern blot analysis of DNA’s isolated from mouse tail biopsy (1–10) and hybridized with the HR1 probe. With help of the *Eco*RI enzymatic digestion, we detect wild type allele at 12.3 kb and targeted allele at 4.5 kb. The 7.6 kb band corresponds to the DNA fragment located outside of the targeting homology (see a). DNA samples 1,3,4,7 and 10 contain correctly targeted ANO1 gene (Ano1^±^). Positions of the size marker (in bp) are shown on the left. (e) Southern blot analysis of DNA’s isolated from mouse DNA’s (1–10) and hybridized with the HR2 probe. This probe detects the presence of the second LoxP site in intron 6. With help of the *Eco*RV enzymatic digestion, we detect the LoxP site in the targeted allele (3.1 kb, see c)

The ANO1 targeting construct (pTMEM16a_targ.) was designed as follows. The 6.8 kb left flanking region containing exons 4–6 (Figure 1a), and intron sequences were PCR amplified from mouse genomic DNA using oligonucleotides TMEM_FAd1 and TMEM_FAr1 (Table S1), and subcloned. The 1.3 kb right flanking region containing intron 7 genomic sequences was PCR amplified using oligonucleotides TMEM_FBd1 and TMEM_FBr1 (Table S1) and subcloned. The 0.5 kb exon 7 genomic region together with intron sequences were PCR amplified using oligonucleotides TMEM_Ex7d1 and TMEM_Ex7r1(Supplementary materials) and consequently subcloned. The exon 7 flanking LoxP site together with the EcoRV site were introduced by PCR cloning with help of the oligonucleotide TMEM_Ex7d1. All individual clones were verified by sequencing and assembled into the final targeting construct in the order depicted on Figure 1b. The pBluescript‐based plasmid backbone together with the negative selection marker (thymidine kinase cassette and diphtheria toxin gene) was added to the right flanking region. The positive selection marker (neomycin cassette flanked by two FRT sites and one LoxP site) was cloned as *Eco*RI – *Bam*HI DNA fragment between right flanking region and 0.5 kb exon 7 genomic PCR clone.

### 2.2 Southern blot DNA probes cloning

The 1.9 kb HR1 probe (outside of the targeted homology, Figure 1c) was PCR amplified from mouse genomic DNA using oligonucleotides TMEM_S1d1 and TMEM_S1r1 (Table S1) and subcloned. The internal 1.2 kb HR2 probe was PCR amplified from the pTMEM16a_targ vector using oligonucleotides TMEM_ISD1 and TMEM_ISR1 (Supplementary materials) and consequently subcloned.

### 2.3 ES cell transfection and selection of targeted clones

CV19 ES cells (passage 13 [129Sv × C57BL/6J]) were expanded in HEPES‐buffered Dulbecco's modified Eagle's medium supplemented with 15% fetal bovine serum (PAA), non‐essential amino acids, L‐glutamine, β‐mercaptoethanol, 1,000 U of recombinant leukemia inhibitory factor (MERCK Millipore, Germany) per ml, and antibiotics (penicillin [100 U/ml] and streptomycin [100 μg/ml]). For electroporation, 2 × 10^7^ cells were resuspended in 0.8 ml Capecchi buffer (20 mM HEPES [pH 7.4], 137 mM NaCl, 5 mM KCl, 0.7 mM Na_2_HPO_4_, 6 mM dextrose, 0.1 mM β‐mercaptoethanol. The targeting vector pTMEM16a_targ was linearized with *Not*I, and 55 μg of DNA was electroporated at 25 μF and 400V in 0.8‐mm electroporation cuvettes (Gene Pulser; Bio‐Rad). After electroporation, cells were cultivated for 10 min at room temperature and plated onto 10 culture dishes containing a gamma‐irradiated monolayer of mouse primary G418‐resistant fibroblast feeder cells. Thirty‐two hours later, 350 µg of G418 (Invitrogen) per ml and 0.2 μM 2’‐deoxy‐2’‐fluoro‐β‐D‐arabinofuranosyl‐5‐iodouracil (FIAU; Moravek Biochemicals and Radiochemicals) were added to the culture medium. The medium was replaced every day, and colonies were picked and analyzed 8 days after plating.

### 2.4 DNA southern blot analysis

Positively targeted ES cell clones were analyzed using the Southern blot DNA method. Approximately 5–10 μg of genomic DNA was digested with *Eco*RI, fractionated on 0.8% agarose gels, and transferred to GeneScreen nylon membranes (NEN DuPont). The membranes were hybridized with a ^32^P‐labeled 1.9‐kb probe containing sequences 5’ to the targeted homology (HR1 probe, Figure 1) and washed with SSPE buffer (0.09 M NaCl, 5 mM NaH_2_PO4, and 0.5 mM EDTA [pH 7.7]) and 0.5% sodium dodecyl sulfate at 65°C. After first screening, correctly targeted event was proved on DNA’s isolated from positively targeted ES cells with *Eco*RI digestion and additionally with *Eco*RV digestion, using a ^32^P‐labeled 1.2‐kb probe containing internal sequences to the targeted homology (HR2 probe, Figure 1a).

### 2.5 Blastocyst injection

Correctly targeted ES cells from two independent clones (2C2 and 3D9) were injected into 3.5‐day B6D2F1 blastocysts. Routinely, we are injecting 12 to 14 ES cells into one blastocoel. After injection, blastocysts were kept in KSOM embryo medium and subsequently transferred into the uteri of 2.5‐day pseudopregnant CD‐1 foster mice. The mice carried pups to term. Chimeras were identified by their agouti coat color contribution. For the germ‐line transmission high percentage male chimaeras were crossed to the C57BL/6J female mice. Heterozygous agouti offspring (*Ano1* ^±^) was confirmed by the Southern blot analysis (Figure 1d,e).

### 2.6 Animals

All mouse procedures were conformed to the guidelines from Directive 2010/63/EU of the European Parliament on the protection of animals used for scientific purposes and performed in compliance with the guidelines for the welfare of experimental animals issued by the Federal Government of Germany. The mouse line was established by breeding male ANO1‐deficient mice with female C57Bl/6J mice to produce heterozygous mice. Pups were weaned at 19 to 23 days after birth, and females were kept separately from males. The mice were housed in standard individually ventilated cages. General health checks were performed regularly in order to ensure that any findings were not the result of deteriorating physical conditions of the animals. All mouse experiments were approved by the Animal Experiments Inspectorate of the Danish Ministry of Environment and Food (nr. 2016‐15‐0201‐00982) and reported in accordance with the ARRIVE (Animal Research: Reporting in vivo Experiments) guidelines.

### 2.7 Solutions

Physiological saline solution (PSS) was used for dissection and myograph experiments. The PSS consisted of (mM) HEPES 10, NaCl 116, KCl 2.82, MgSO_4_ 1.2, NaHCO_3_ 25, KH_2_PO_4_ 1.18, CaCl_2_ 1.6, EDTA 0.03 and glucose 5.5. The PSS was warmed to 37°C and bubbled with 5% CO_2_ in air to reach pH 7.4.

### 2.8 Functional studies in vitro

The mice were sacrificed at the age of 1.5–4 months. Two‐millimeter long segments of the abdominal aorta, saphenous and tail arteries were isolated in ice‐cold PSS and mounted in an isometric wire myograph (Danish Myo Technology). Arteries were normalized by first finding the internal circumference that corresponds to transmural pressure of 100 mmHg (IC_100_). Second, artery diameter was set to correspond to 90% of IC_100_ (Mulvany & Halpern, 1977). Force was recorded with a PowerLab 4/25 – Chart7 acquisition set up (ADInstruments Ltd.). The results of force measurement are expressed as wall tension in N/m (Tension = Force/ [2 segment length]). To assess the role of Ca^2+^ release, in some experiments arteries were incubated for 5 min in Ca^2+^‐free bath solution prior to 10 µM noradrenaline stimulation. When indicated, endothelium was denuded by blowing for 1 min luminal air through the arteries mounted in the myograph, and then, tested by disappearance of vasorelaxation to acetylcholine.

Measurements of intracellular Ca^2+^ in smooth muscle cells and wall tension in myograph were made simultaneously (Jensen et al., 1992; Peng et al., 1998). Arterial segments were loaded with 2.5 µM fura 2‐acetoxymethyl ester dissolved in DMSO with 0.1% (wt/vol) cremophor and 0.02% (wt/vol) pluronic F127 for 2h. Arteries were excited by a 75W xenon light source alternately at 340 and 380 nm, and emitted light was measured at 515 nm. Background fluorescence was determined and subtracted from obtained measurements. Fluorescence was collected using Felix32 software (version 1.2, Photon Technology). Intracellular Ca^2+^ was expressed as the ratio of fluorescence during excitation at 340 and 380 nm.

### 2.9 Morphology measurements

Media and wall thickness of the arteries mounted in the myograph were measured with a light microscope (40× water immersion lens) as previously described (Mulvany et al., 1978). Media thickness was normalized to a diameter corresponding to 90% of IC_100_ assuming a constant media volume.

### 2.10 Western blot

Arteries were isolated, frozen in liquid nitrogen and stored at −80°C. Samples were lysed in ice‐cold lysis buffer (Pierce, Thermo Scientific) containing and Halt Protease inhibitor cocktail 1:100 (Thermo Scientific) using a pellet pestle. The homogenate was sonicated for 45 s, centrifuged at 13,000 rpm for 10 min and the supernatant collected. Total protein content in the samples was quantified using a BCA Protein Assay kit (Pierce, Thermo Scientific). A mix of 10 parts of 4x Laemmli sample buffer (Bio‐rad) and one part 2 M dithiothreitol was added to the protein samples containing 10 μg of protein to raise the sample volume to 20 μl. Afterward, samples were heated to 70°C for 10 min. Twenty microliters of sample was loaded on 4%–20% precast polyacrylamide stain‐free gels (CriterionTM TGX Stain‐freeTM precast gel, BioRad). Total protein load was detected on the stain‐free gels using UV‐light in imaging system (c600, Azur Biosystems). Separation was performed using electrophoresis. Proteins were transferred to PVDF‐membranes (Merck Millipore). PVDF membranes were cut in half at approximately 75 kDa. The top part of the PVDF‐membrane was blocked for 2 hr with 5% nonfat dry milk (Blotting‐Grade, Bio‐Rad) in Tris‐buffered saline with 0.1% Tween. The bottom part of the membrane was blocked for 2 hr with 5% bovine serum albumin (Sigma Aldrich) in Tris‐buffered saline with 0.1% Tween. Afterwards membranes were incubated overnight at 4°C in blocking buffer with primary antibody. The top part was incubated with either antibody against ANO1 (1:250, ab53212; Abcam) or L‐type Ca^2+^ channels (1:500, #ACC‐003; Alomone) and the bottom part was incubated with pan‐actin antibody (1:2000, #4968; Cell Signaling). Next day membranes were incubated with HRP‐conjugated secondary goat‐anti‐rabbit antibody (1:2000 Cell Signaling Technology) for 2 hr. Detection was performed using ImageQuant LAS 4000 (GE Healthcare Life Sciences). Equal loading was confirmed using pan‐actin band at 45 kDa. Quantification was made using ImageJ (v1.48, NIH). Except some preliminary experiments where protein expression was semi‐quantified as a ratio of intensity of bands for ANO1 and pan‐actin proteins, detected protein was normalized as a ratio to total protein load measured for the same probe. Analyses were done using the ImageJ program (NIH). The normalized protein ratio is shown in percentage of mean ratio in the wild type group.

### 2.11 Single cell voltage‐clamp

The voltage‐clamp measurements of the Ca^2+^‐activated Cl^−^ current were made as previously described (Matchkov et al., 2008). Shortly, dissected tail arteries were stored overnight at 4°C in a solution for enzymatic digestion, in mM: NaCl 110, KCl 5, MgCl_2_ 2, KH_2_PO_4_ 0.5, NaH_2_PO_4_ 0.5, NaHCO_3_ 10, CaCl_2_ 0.16, EDTA 0.49, Na‐HEPES 10, glucose 10, taurine 10 at pH 7.0, as well as 1.5 mg/ml papain, 1.6 mg/ml albumin, and 0.4 mg/ml dl‐dithiothreitol. Next day, the arteries were incubated in this solution for 5–10 min at 37°C and single cells were released by trituration with a polyethylene pipette into the experimental solution. Isolated cells were used within next 4 hr.

All experiments were made at room temperature (22–24°C). Recordings were made with an Axopatch 200B amplifier (Axon Instruments, Inc.) in whole‐cell configuration. Data were sampled at a rate of 2 kHz and filtered at 1 kHz. Data acquisition was done with the software package Clampex 7 for Windows (Axon Instruments, Inc.) and analyzed using Chart8 (ADInstruments Ltd.). The Ca^2+^‐activated currents were evoked by patch pipette solution containing the following (in mM): 140 CsCl, 5.5 Ca(OH)2, 0.1 MgATP, 6 EGTA, and 10 HEPES, at pH 7.35 (free Ca^2+^ concentration 900 nM, estimated using WEBMAXC v. 2.22 [Chris Patton, Stanford University]). The bath solution contained the following (in mM): 140 CsCl, 0.1 CaCl2, and 10 HEPES, at pH 7.4. Membrane potential was held at −60 mV. The current–voltage (I–V) relationship was determined by stepping the potentials (20 mV increments) between −60 and +60 mV. The Ca^2+^‐activated Cl^−^ current was then inhibited by 10 µM T16A_inh_‐A01 (Tocris Bioscience), and the Ca^2+^‐activated Cl^−^ current was isolated by subtraction of the current before and after T16A_inh_‐A01 application. Currents were normalized to cell capacitances.

### 2.12 Relative protein quantification using 10‐plex tandem mass tags (TMT)

Tail artery segments were dissected and lysed as described above for Western blot protocol. Proteins were isolated by acetone precipitation, re‐dissolved in 0.2 M TEAB (Triethylammonium bicarbonate) followed by reduction by dithiothretiol (DTT, 5 mM for 30 min at 50°C), alkylation by iodoacetamide (IAA, 15 mM for 30 min at room temperature in the darkness), and proteolytic cleavage by the addition of trypsin in a 1:50 trypsin:protein ratio and overnight incubation at 37°C. The resulting peptide samples were labelled with two sets of tandem mass tags from a 10‐plex TMT set using the mass tags 127N, 127C, 128N, 128C, 129N, 129C, and 131. A pool of all samples was labelled with mass tag 126 and served as reference channel. Tagged peptide samples were mixed and subsequently fractionated by high‐pH reversed phase liquid chromatography and analyzed by reversed phase nano‐liquid chromatography tandem mass spectrometry (RP‐nanoLC‐MSMS). In brief, the tagged peptide samples were combined, lyophilized, purified, and fractionated into five fractions by high pH liquid chromatography virtually as previously described (Mulorz et al., 2020). Fractions were analyzed in triplicates by RP‐nanoLC‐MS/MS analysis on an Orbitrap Eclipse mass spectrometer (Thermo Fisher Scientific) equipped with a nanoHPLC interface (Dionex UltiMate 3000 nano HPLC). Briefly, samples were separated also as previously described (Mulorz et al., 2020). Mass spectra of eluting peptides were acquired in positive ion mode applying automatic data‐dependent switch between an Orbitrap survey MS scan in the mass range of 400–1,200 m/z followed by peptide fragmentation applying a normalized collisional energy of 40% in a 3‐s duty cycle. The AGC target was set to “standard” at a resolution of 60,000 at m/z 200 and 200,000 ions at a resolution of 30,000 with turbo‐TMT setting at m/z 200 for MS/MS scans. Ion selection threshold was set to 25,000 counts. Selected sequenced ions were dynamically excluded for 60 s. All raw data files were processed and quantified using Proteome Discoverer version 2.4 (Thermo Scientific) also as previously described (Mulorz et al., 2020).

### 2.13 Ingenuity pathway analysis

A list of TMT quantified proteins was uploaded to Ingenuity Pathway Analysis software (Qiagen) for functional interpretation. The proteins, which expression differed significantly between genotypes, were identified and their association with cardiovascular disease and function was suggested. Z‐scores predict potential directional changes for identified conditions by using information about the direction of protein expression changes and based on comparison with a model that assigns random regulation directions. A negative *z*‐score indicates suppression of that pathway, while a positive *z*‐score indicates enhancement.

### 2.14 Blood pressure measurements

Blood pressure was measured using radiotelemetry as described previously (Jensen et al., 2018). Mice were anesthetized by a combination of ketamine (33 mg/100 g) and xylazine (7.5 mg/100 g) and placed on a thermostatically controlled warming platform to maintain body temperature at 37°C. A midline incision through the shaved skin on the neck was made, and the mandibular glands were separated to access the carotid artery. The catheter of a HD‐X11 radiotelemetry transmitter (Data Sciences International) was placed into the carotid artery and the transmitter body was placed in a subcutaneous pocket. The skin incision was closed using 6‐0 non‐absorbable suture and a painkiller (0.2 ml/kg; Temgesic; Schering‐Plough Europe) was injected subcutaneously at the end of operation. Mice were allowed to recover for at least 1 week before measurements were started. Telemetry signals were recorded at 256 Hz in 10‐s intervals each minute. Registration was performed with Dataquest A.R.T software 4.3. Analyses were performed with Ponemah 8 (Data Sciences International). Arterial pressure was averaged at midday (between 11 a.m. and 1 p.m.) and midnight (between 11 p.m. and 1 a.m. next day) for each group. To assess the effect of adrenergic activation, phenylephrine (5 mg/kg) dissolved in the vehicle solution containing 0.9% NaCl was injected into the peritoneum.

### 2.15 Statistical analysis

Statistical analysis was performed using Graph Pad Prism 8.0. Concentration‐response curves were fitted to experimental data using four‐parameter, non‐linear regression curve fitting. From these curves, pD_2_ (‐log to the concentration required to produce a half‐maximal response) and maximal response were derived and compared using an extra sum‐of‐squares *F* test. Statistical comparison of other values was performed using Student *t* test. All values are given as mean ± standard deviation. Significance was assumed if *p* < .05 and *n* refers to number of arteries, only one of each type of artery was investigated from each mouse.

## 3 RESULTS

### 3.1 Heterozygote ANO1 knockout mice had reduced ANO1 protein expression in the vascular wall of different arteries

**FIGURE 2:**
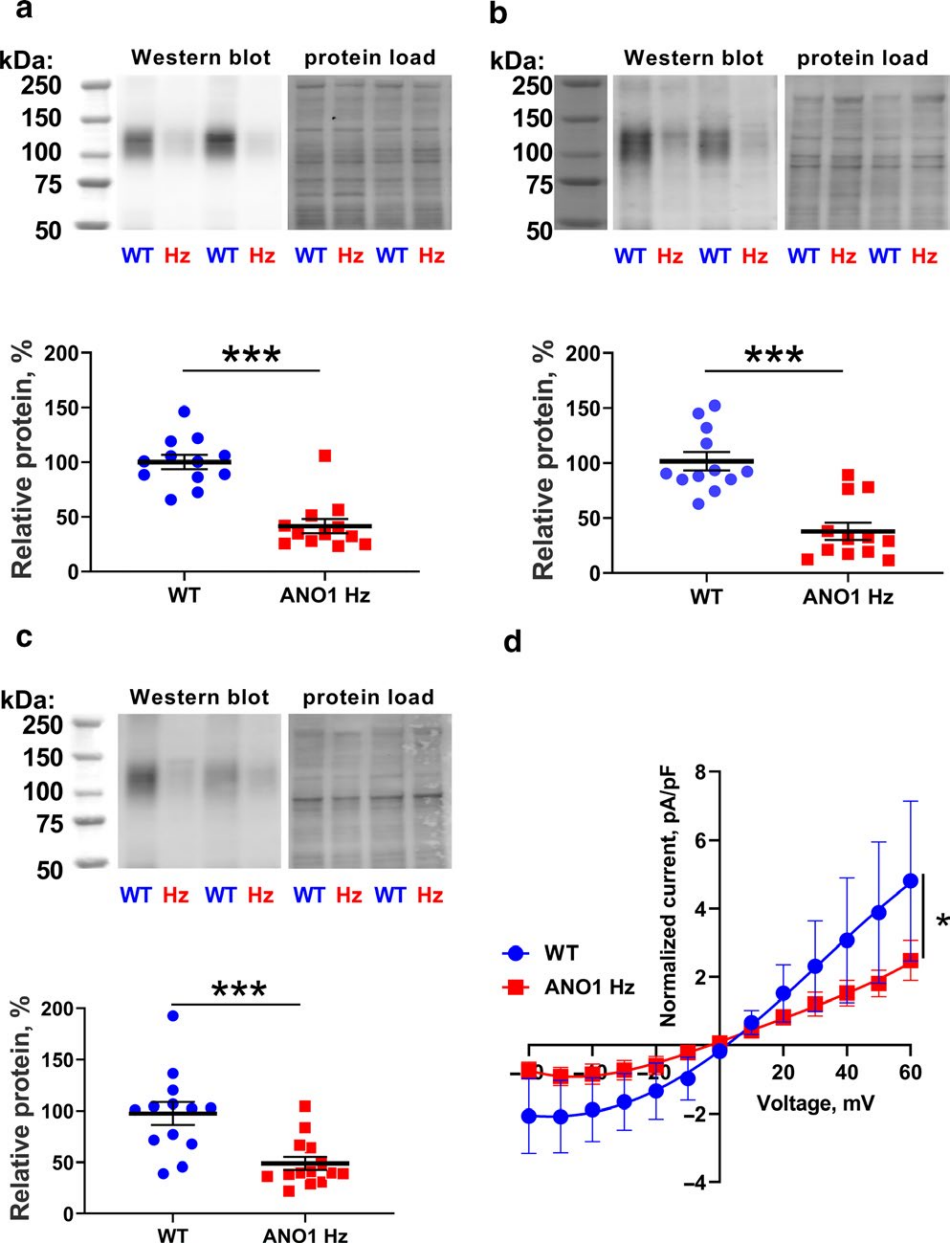
ANO1 protein expression is reduced in the vascular wall of ANO1 heterozygous mice. Western blot (representative blots in upper panels; from left to the right: molecular weight marker, Western blot and protein load) suggest reduced expression level of ANO1 protein in aorta (a; *n* = 12), tail (b; *n* = 12) and saphenous (c; *n* = 13–14) arteries from ANO1 heterozygous mice (ANO1 Hz) in comparison with wild type (WT). * and **p < .05 and .01 (unpaired *t* test). Voltage‐dependence of T16Ainh‐A01 sensitive current in smooth muscle cells isolated from tail arteries of ANO1 Hz and WT mice. *p < .05, *n* = 6 (*F*‐test)

To assess the expression of ANO1 in heterozygous mice, Western blot‐based semi‐quantification analyses were performed. The ANO1 protein expression in aorta from ANO1 heterozygous mice was significantly lower than protein expression in aorta from wild type (Figure 2a). Although the ANO1‐specific band intensity in wild type mice was lower in saphenous and tail arteries compared to aorta, ANO1 expression was also reduced in these arteries from ANO1 heterozygous mice compared to wild type mice (Figure 2b,c).

### 3.2 ANO1 knockdown suppressed the Ca^2+^‐activated Cl^−^ current in smooth muscle cells

Smooth muscle cells isolated from tail arteries of ANO1 heterozygous knockout mice had significantly smaller T16A_inh_‐A01 sensitive membrane conductance than wild type smooth muscles (Figure 2d). The results suggest approximately 50% reduction in the Ca^2+^‐activated Cl^−^ current in ANO1 downregulated smooth muscle cells from tail arteries.

### 3.3 Changes in vasoconstriction in response to agonists and K^+^‐induced depolarization of arteries with reduced expression of ANO1 depends on the type of blood vessel

**FIGURE 3:**
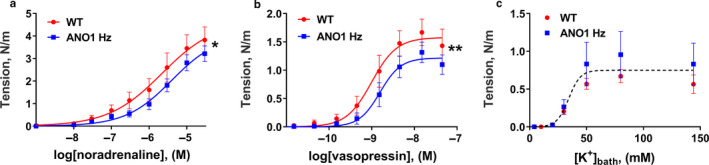
Knockdown of ANO1 protein reduced agonist‐induced contraction aorta. Concentration‐response curves to noradrenaline (a), vasopressin (b) and elevation bath K^+^ (c) for aorta segments dissected from ANO1 heterozygous (ANO1 Hz) and wild type (WT) mice. *n* = 5. * and **p < .05 and .01 (extra sum‐of‐squares *F* test)

Aorta from ANO1 heterozygous mice had smaller wall tension development than wild type aorta in response to concentration‐dependent elevation of both noradrenaline (Figure 3a) and vasopressin (Figure 3b). No difference in contractile response to increasing K^+^ concentration was seen between the two groups (Figure 3c).

**FIGURE 4:**
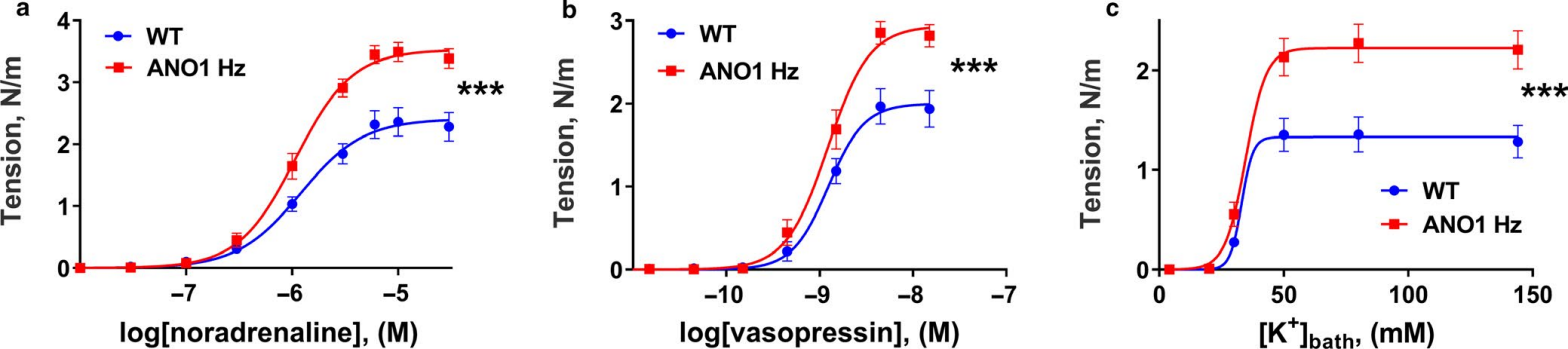
Knockdown of ANO1 protein potentiated contractile responses to agonist stimulation and K^+^‐induced depolarization of tail arteries. Concentration‐response curves to noradrenaline (a), vasopressin (b), and elevation of bath K^+^ (c) for tail arteries from ANO1 heterozygous (ANO1 Hz) and wild type (WT) mice. *n* = 13–15. ***p < .001 (extra sum‐of‐squares *F* test)

**FIGURE 5:**
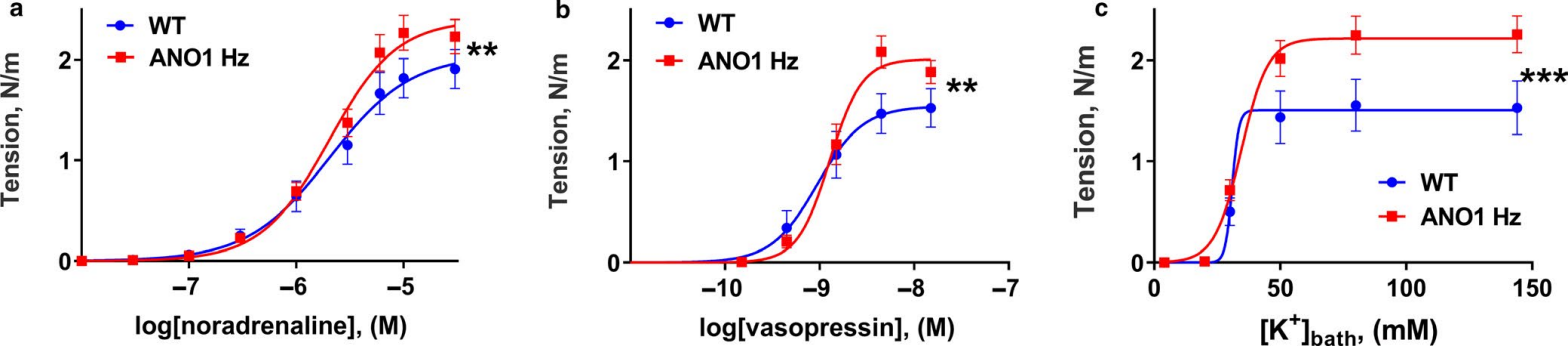
Increased contraction to agonist stimulation and K^+^‐induced depolarization of saphenous arteries from ANO1 heterozygous mice. Concentration‐response curves to noradrenaline (a), vasopressin (b) and elevation of bath K^+^ (c) for saphenous arteries from ANO1 heterozygous (ANO1 Hz) and wild type (WT) mice. *n* = 12 – 13. ** and ***p < .01 and .001 (extra sum‐of‐squares *F* test)

In contrast to aorta, force development in response to noradrenaline, vasopressin, and K^+^ was significantly increased in tail arteries from ANO1 heterozygous mice in comparison with wild type (Figure 4a–c). Similarly, saphenous arteries from ANO1 heterozygous mice also showed increased contraction to noradrenaline, vasopressin, and K^+^ compared to wild type group (Figure 5a–c).

### 3.4 An abnormal potentiation of contraction of tail arteries knocked down for ANO1 is not due to endothelial dysfunction

**FIGURE 6:**
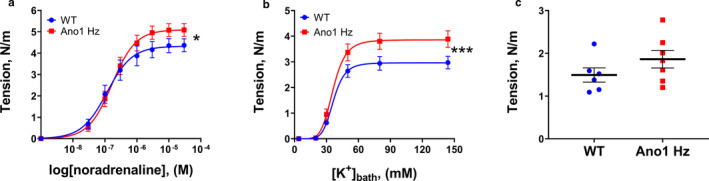
Increased contraction of tail arteries knocked down for ANO1 is not due to endothelial dysfunction nor abnormal release of intracellular Ca^2+^. Endothelium‐denuded tail arteries were constricted with noradrenaline (a) and elevation of bath K^+^ (b). *n* = 6–7. * and ***p < .05 and .001 (extra sum‐of‐squares *F* test). Application of 10 µM noradrenaline in Ca^2+^‐free bath solution produced similar contraction of wild type and ANO1‐downregulated tail arteries (c; *n* = 6–7)

Endothelium is an important contributor to control of vascular tone. To test whether endothelial dysfunction is responsible for the increased contraction of tail arteries from ANO1 heterozygous mice, we compared their contractile responses after endothelium removal. The lack of functional endothelium was assessed by the absence of relaxation in response to 10^–5^ M acetylcholine. Endothelium‐denuded tail arteries from ANO1 heterozygous mice contracted stronger to both noradrenaline (Figure 6a) and K^+^‐induced depolarization (Figure 6b).

### 3.5 Ca^2+^ release is increased in tail arteries from ANO1 heterozygous mice

Noradrenaline (10 µM) applied in a Ca^2+^‐free bath solution produced similar contractile responses in tail arteries from wild type and ANO1 heterozygous mice (Figure 6c). This suggests that ANO1 heterozygous mice have unchanged agonist‐induced Ca^2+^ release from intracellular stores.

### 3.6 Morphology measurements did not reveal any difference in arterial structure of ANO1 heterozygous and wild type mice

**FIGURE 7:**
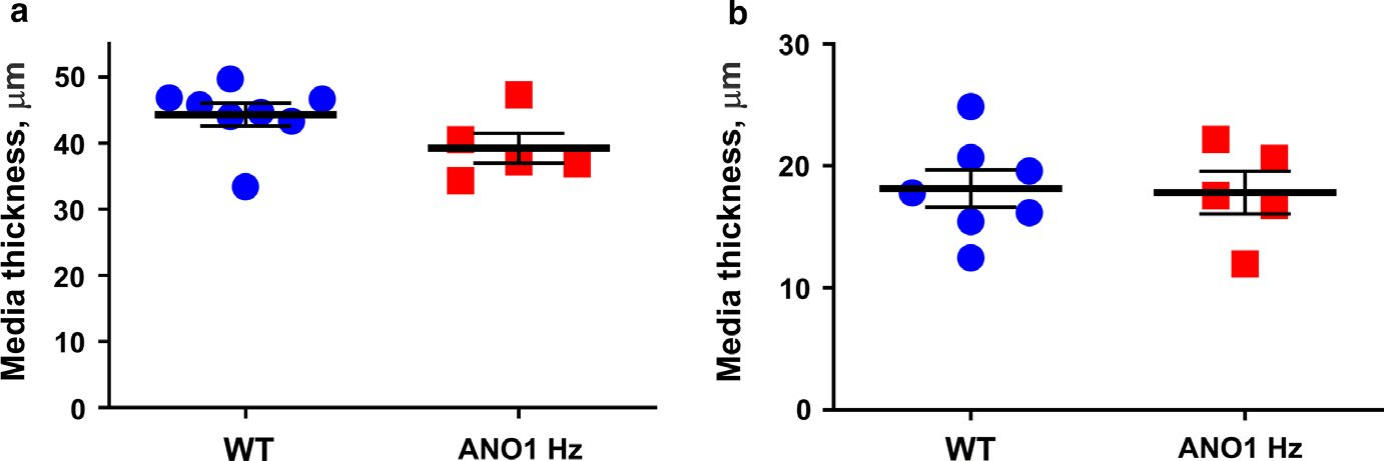
No difference in media thickness of saphenous and tail arteries from ANO1 heterozygous and wild type mice. The measurements of media thickness of tail (a; *n* = 5–8) and saphenous (b; *n* = 5–7) arteries did not find any difference between ANO1 heterozygous and wild type mice (Student *t* test)

The increased contractility of arteries from ANO1 heterozygous mice could be explained by an increased media thickness. To test this possibility, we measured media thickness of the arteries. Media thickness in tail (Figure 7a) and saphenouos (Figure 7b) arteries did not differ between groups.

### 3.7 The increased agonist‐induced contraction of saphenous arteries from ANO1 heterozygous mice was associated with increased intracellular Ca^2+^ response

**FIGURE 8:**
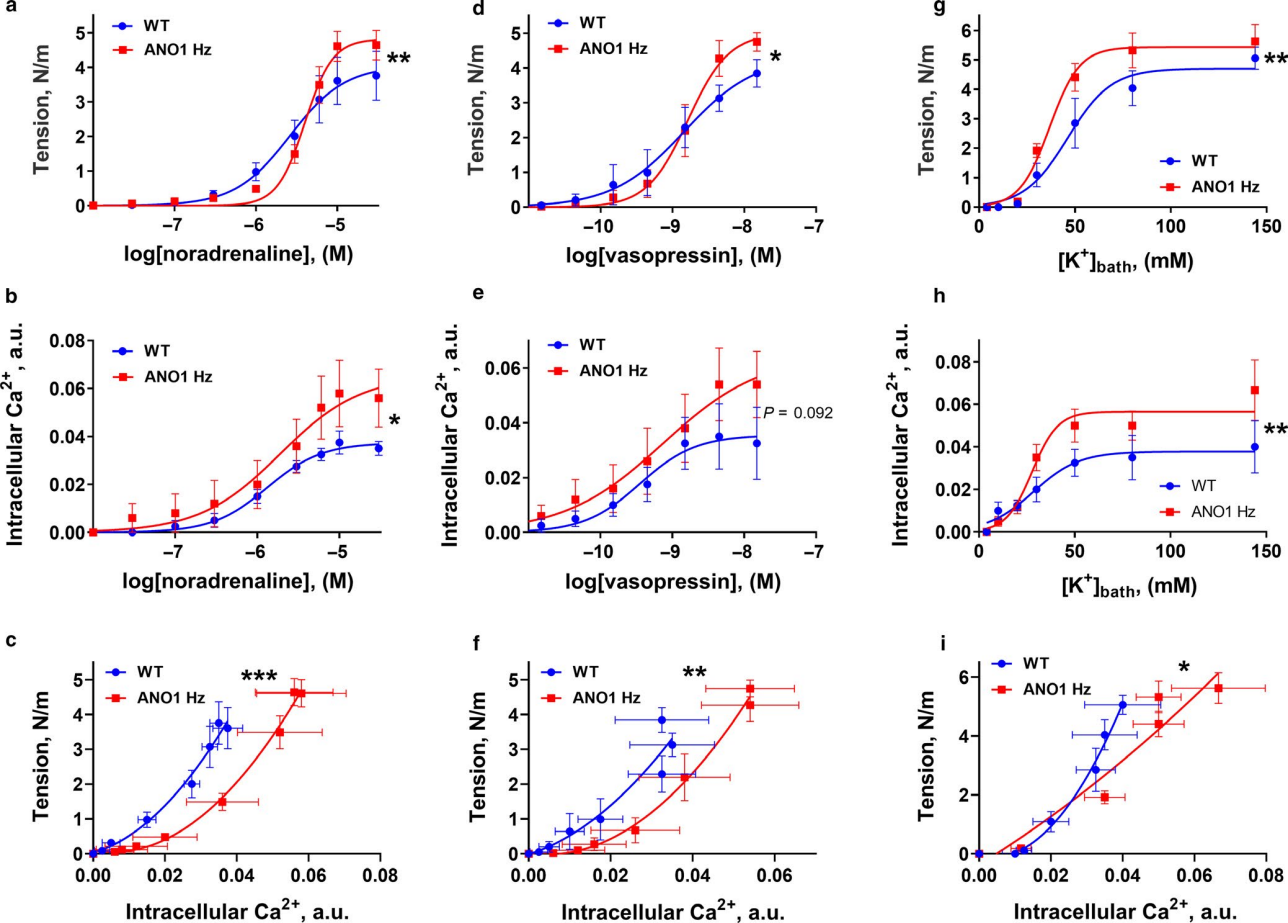
An increased contractile response of saphenous artery from ANO1 heterozygous mice is mediated by elevated voltage‐gated Ca^2+^ influx in spite of reduced Ca^2+^ sensitivity of smooth muscle cells. Simultaneous recordings of contraction (a, d, g) and intracellular Ca^2+^ (b, e, h) in saphenous artery from ANO1 heterozygous and wild type mice in response to noradrenaline (a, b), vasopressin (d, e) and K^+^‐induced depolarization (g, h). To assess the sensitivity of smooth muscle cells to intracellular Ca^2+^, wall tension was plotted as a function of intracellular Ca^2+^ changes in response to noradrenaline (c), vasopressin (f), and K^+^‐induced depolarization (i). *, ** and ***p < .05, .01 and .001 (extra sum‐of‐squares *F* test)

Simultaneous measurements of arterial contraction and intracellular Ca^2+^ changes in response to increasing concentrations of noradrenaline showed that both force and intracellular Ca^2+^ increase were enhanced in arteries from ANO1 heterozygous mice (Figure 8a,b). Similar potentiation of contraction and intracellular Ca^2+^ elevation was also seen in response to vasopressin, though intracellular Ca^2+^ response increase did not achieve statistical significance (*p* = .092; Figure 8d,e). Importantly, when wall tension was plotted as a function of intracellular Ca^2+^, arteries from ANO1 heterozygous mice had a reduced contraction at the same Ca^2+^ concentration in comparison with wild type, that is, ANO1 downregulated smooth muscles were less sensitive to intracellular Ca^2+^ changes. These wall tension – intracellular Ca^2+^ relations were nearly identical for stimulation with noradrenaline and vasopressin (Figure 8c,f). Moreover, when the arterial wall was depolarized with increasing concentrations of bath K^+^, an increased contractile response of ANO1 downregulated arteries (Figure 8g) was also accompanied with increased intracellular Ca^2+^ elevation (Figure 8h) and reduced sensitivity to intracellular Ca^2+^ (Figure 8c,f,i).

### 3.8 No difference in vascular L‐type Ca^2+^ channel expression between ANO1 heterozygous mice and wild type

**FIGURE 9:**
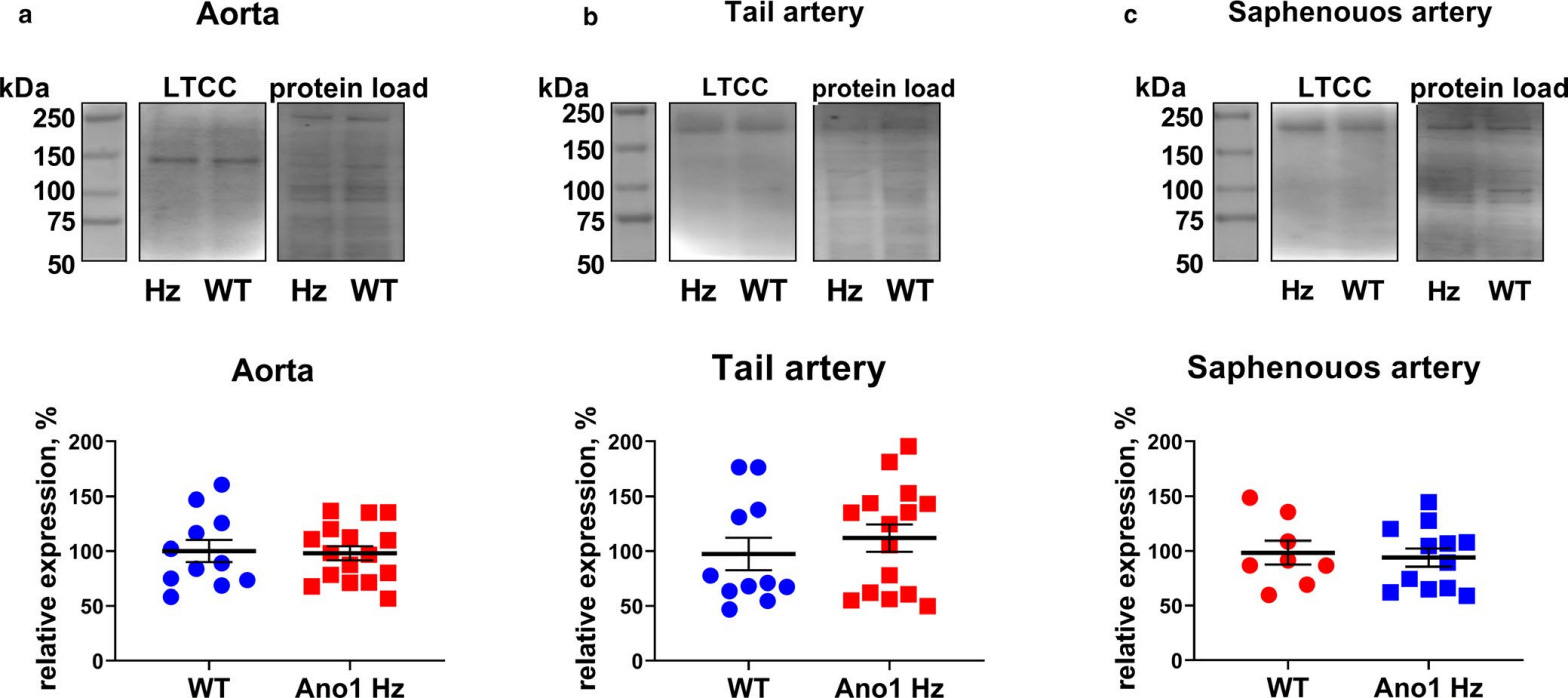
The expression of L‐type Ca^2+^ channels (LTCC) is the same in arteries from ANO1 heterozygous mice (Hz) and wild type controls (WT). LTCC expression was compared in aorta (a; *n* = 11–16), tail arteries (b; *n* = 11–15), and saphenous arteries (c; *n* = 8–15). Upper panel shows the representative image of (from left to the right) molecular weight marker, Western blot and protein load, lower panel shows averaged data

We next asked whether the enhanced intracellular Ca^2+^ increase in small arteries from ANO1 knockdown mice is a result of increased L‐type Ca^2+^ channel expression. No difference in the expression of L‐type Ca^2+^ channels between ANO1 downregulated and wild type arteries was found in aorta (Figure 9a), tail arteries (Figure 9b), and saphenous arteries (Figure 9c). Interestingly, the molecular weight of the L‐type Ca^2+^ channels detected in tail and saphenous arteries was significantly higher than those in aorta. Different splice variants of the L‐type Ca^2+^ channels have previously been reported in vascular smooth muscle cells (Bannister et al., 2011) but it cannot explain the difference in contraction and Ca^2+^ responses between genotypes of the same type of artery seen in this study.

### 3.9 Ingenuity pathway analysis suggested functional changes in vascular smooth muscle cells from tail arteries of ANO1 heterozygous mice

**FIGURE 10:**
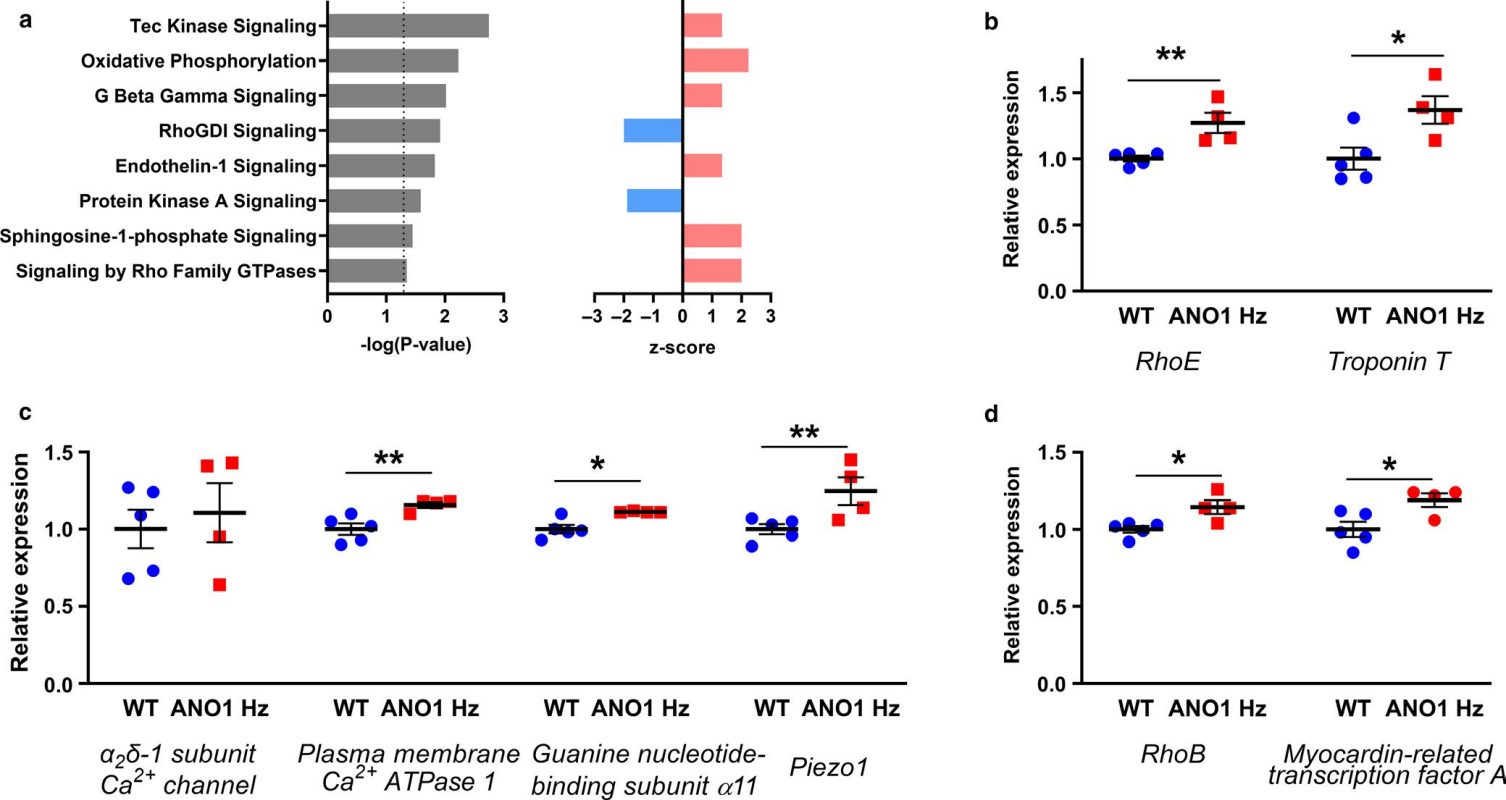
Ingenuity Pathway Analysis suggests changes in vascular of tail arteries from ANO1 heterozygous mice. Proteomics of tail arteries from ANO1 heterozygous (*n* = 4, where three mouse arterial lysates where pulled into one probe, while other three probes were each from a single mouse) and wild type mice (*n* = 5 mice). (a) Canonical pathways affected by ANO1 knockdown and related to vascular metabolism and function as suggested by Ingenuity Pathway Analysis and listed against their significance (left) and *Z*‐scores (right). *Z*‐scores predict either suppression (blue) or potentiation (red) of cardiovascular condition in ANO1 knockdown compared to wild type. For details see Table S3. (b) Differentially expressed Rho‐related GTP‐binding protein RhoE suggested reduced Ca^2+^ sensitization while Troponin T suggested potentiation of contraction. (c) No difference in the expression of auxiliary α2δ‐1 subunit of voltage‐dependent Ca^2+^ channels (Bannister et al., 2009, 2012)was found, while increased expression of the plasma membrane Ca^2+^ ATPase 1, guanine nucleotide‐binding protein α11 subunit and Piezo1 protein was found in the tail arteries from ANO1 heterozygous mice. (d) Rho‐related GTP‐binding protein RhoB that is involved in hypoxia‐induced vascular responses and a potent coactivator of smooth muscle contractile genes, myocardin‐related transcription factor A were upregulated in the tail arteries from ANO1 heterozygous mice. * and **p < .05 and <.01. For details about involved proteins see Table S2

Proteomics data of tail arteries from ANO1 heterozygous and wild type mice were analyzed to assess the pathways underlying the observed functional changes. We identified 3,051 mapped proteins, thereof 228 significantly (*p* < .05) upregulated and 13 significantly (*p* < .05) downregulated (Table S2). The following analysis suggested significant changes in several canonical pathways related to vascular metabolism and function (Figure 10a; Table S3).

Detailed analyses of differentially mapped proteins (Table S2) supported our other observations. We found an increased expression of Rho‐related GTP‐binding protein RhoE that inhibits on Rho‐associated protein kinase (ROCK) and Ca^2+^‐sensitization in vascular smooth muscle cells (Loirand et al., 1999; Shimokawa et al., 2016; Figure 10b). Troponin T, which contributes to smooth muscle contraction, (Kajioka et al., 2012) was also upregulated in the tail arteries from ANO1 heterozygous mice (Figure 10b). In addition to the unchanged expression of the L‐type Ca^2+^ channels (Figure 9), no difference in the expression of auxiliary α2δ‐1 subunit of these voltage‐dependent Ca^2+^ channels (Bannister et al., 2009, 2012) was found (Figure 10c). There was however, an increased expression of the plasma membrane Ca^2+^ ATPase may be compensatory in relation to the increased Ca^2+^ responses (Figure 10c). The Ca^2+^ elevation maybe a result of an upregulation of guanine nucleotide‐binding protein α11 subunit and Piezo1 channel protein in the tail arteries from ANO1 heterozygous mice (Figure 10c). Finally, Rho‐related GTP‐binding protein RhoB and myocardin‐related transcription factor A were upregulated in the tail arteries from ANO1 heterozygous mice (Figure 10d).

### 3.10 No difference in blood pressure control between ANO1 heterozygous and wild type mice

**FIGURE 11:**
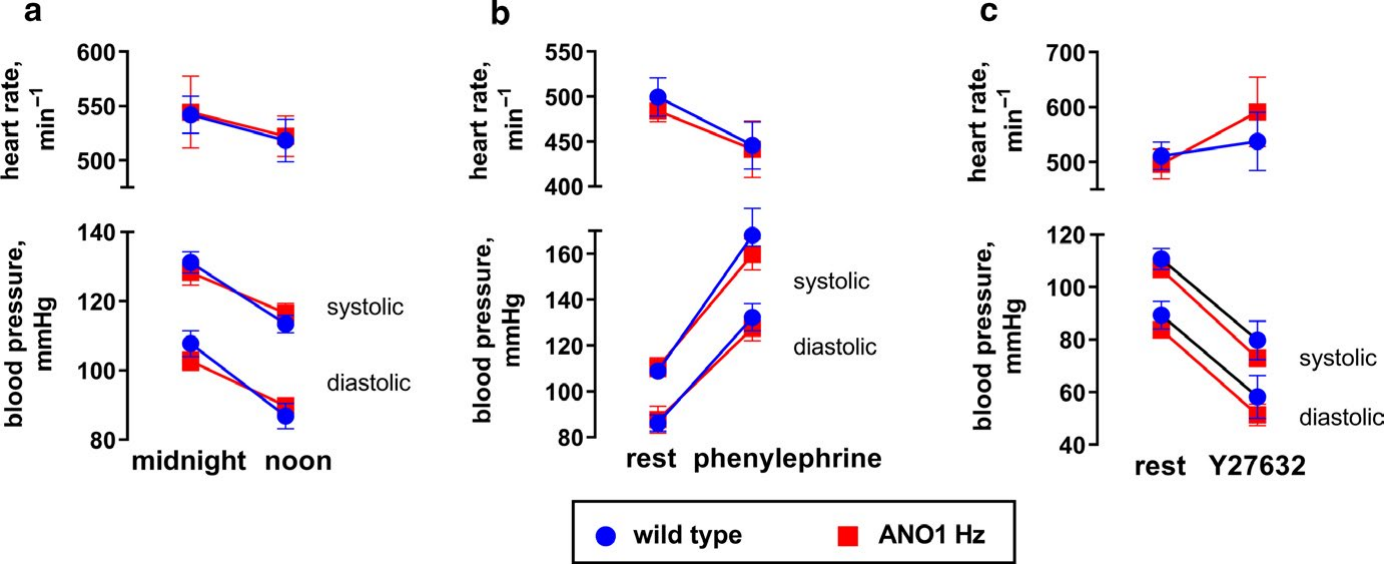
No difference between blood pressure and heart rate of ANO1 heterozygous and wild type mice. Radiotelemetry recordings of blood pressure and heart rate demonstrated circadian rhythm and no difference between genotypes (a). Challenge with an acute injection of 5 mg/kg phenylephrine elevated blood pressure and reduced heart rate (b), no difference between the groups was seen. Y27632 (10 mg/kg) injection reduced blood pressure and elevated heart rate to the same extend in ANO1 heterozygous and wild type mice (c). *n* = 8

Blood pressure and heart rate were continuously recorded in mice using radiotelemetry. Both wild type and ANO1 heterozygous mice had circadian rhythm variations in arterial pressure and heart rate that is consistent with murine nocturnal behavior (Figure 11a). No significant differences in mean, systolic and diastolic blood pressures were found between the groups.

To test whether the increased contractility to high concentrations of agonists of the saphenous and tail arteries in ANO1 heterozygous mice was reflected in vivo we challenged the mice with phenylephrine. However, a single i.p. injection of phenylephrine (5 mg/kg) caused the same elevation of blood pressure and associated reduction of heart rate in the two groups (Figure 11b). Finally, we tested the hypotensive effect of Rho kinase inhibitor, Y27632. Injection of 10 mg/kg Y27632 had the same acute hypotensive effect and heart rate increase in ANO1 heterozygous and wild type mice.

## 4 DISCUSSION

We developed a mouse with knockout of exon 7 in the ANO1 gene on one of the alleles. Consistent with the genetic make‐up of these mice, the protein level in the three arteries we investigated was about 50% of the wild type level. We also found an approximately 50% reduction to in the Ca^2+^‐activated Cl^−^ conductance in tail artery smooth muscle cells from ANO1 knockdown mice, which is in accordance with the importance of ANO1 protein for this conductance (Caputo et al., 2008; Schroeder et al., 2008; Yang et al., 2008). These mice allowed us to study the role of ANO1 for vascular function avoiding problems associated with lethality of mice with homozygous knockout of ANO1 (Rock et al., 2008) and unspecific effects of siRNA (Bulley et al., 2012; Dam, et al., 2014) and with the lack of specificity of Cl^−^ channel inhibitors (Boedtkjer et al., 2015; Greenwood & Leblanc, 2007; Rock & Harfe, 2008).

We found a decrease in responses of aorta from the ANO1 heterozygous mice to noradrenaline and vasopressin and no change in the response to K^+^‐induced depolarization. Since the Ca^2+^‐activated Cl^−^ conductance mediated by ANO1 contributes to membrane depolarization (Askew Page et al., 2019; Dam, et al., 2014), knockdown of ANO1 is expected to reduce depolarization and hence force development, and the data from aorta are consistent with this. The Ca^2+^‐activated Cl^−^ channels are proposed to be a key step in agonist‐induced constriction of vascular smooth muscle cells (Bulley & Jaggar, 2014; Dam, et al., 2014; Large & Wang, 1996; Leblanc et al., 2005, 2015; Matchkov et al., 2013, 2015). It has been suggested that Ca^2+^‐activated Cl^−^ efflux depolarizes the membrane and potentiates voltage‐dependent Ca^2+^ influx, which would further stimulate the Ca^2+^‐activated Cl^−^ channels. This was supported by the studies with expressional manipulations and pharmacological inhibition of ANO1 (Bulley et al., 2012; Dam, et al., 2014; Davis et al., 2013; Jensen et al., 2018). The lack of ANO1 role in vasoconstriction induced by K^+^ depolarization further supports this hypothesis. This was shown for aorta where ANO1 was downregulated by constitutive siRNA expression (Jensen et al., 2018) or knocked out (Heinze et al., 2014) and in cerebral small arteries (Bulley et al., 2012). However, siRNA‐induced downregulation of ANO1 in rodent small arteries (i.e., tail and mesenteric arteries) suppresses both agonist‐induced contraction and responses to K^+^‐induced depolarization (Dam, et al., 2014; Jensen et al., 2018). In these cases, the expression of voltage‐gated Ca^2+^ channels was reduced, which might explain the reduced response to K^+^‐induced depolarization, and may suggest a close interaction between ANO1 and L‐type Ca^2+^ channels.

To our surprise, we observed an increased contraction to noradrenaline and vasopressin of tail and saphenous arteries from ANO1 heterozygous mice compared to arteries from wild type mice. Thus, it is unlikely that the enhanced response is due to upregulation of a specific receptor. Moreover, the response to K^+^‐induced depolarization was also increased, making it unlikely that the enhanced contractile responses to the agonists are due to increased depolarization of smooth muscle cells. The increased responses to both agonist stimulation and K^+^‐induced depolarization could be explained by an increased media thickness. However, there was no difference in media thickness between the groups. Moreover, endothelium‐denuded tail arteries from ANO1 heterozygous mice also constricted stronger than endothelium‐denuded tail arteries from wild type mice in response to noradrenaline and K^+^‐induced depolarization. This suggests that the changed endothelial function is not responsible for the observed genotype difference in small artery responses.

We next investigated whether the increased contractility could be due to increased intracellular Ca^2+^ activity or to an increased sensitivity of smooth muscle cells to intracellular Ca^2+^. We found that increased agonist‐induced contraction was associated with elevated Ca^2+^ changes. Since there was no difference in resting intracellular Ca^2+^ (estimated as the fluorescence ratio; not shown) in the vascular wall prior to stimulation, the difference in Ca^2+^ responses most likely reflects an increased Ca^2+^ concentration. Moreover, we found potentiated intracellular Ca^2+^ changes and contraction in response to K^+^‐induced depolarization. This suggests that increased contraction of tail and saphenous arteries might be a result of increased voltage‐dependent Ca^2+^ influx. A close interaction between ANO1 and L‐type Ca^2+^ channels is previously shown in retina (Caputo et al., 2015), the anal sphincter (Zhang et al., 2016), and in cardiomyocytes (Horvath et al., 2016). However, no difference in the expression of voltage‐gated L‐type Ca^2+^ channels between arteries from ANO1 knockdown and wild type mice was found in this study. Whether there is an effect of ANO1 on the voltage‐gated Ca^2+^ channels at the regulatory (e.g., changing the channel opening dynamic) level remains to be studied. However, the expression of the auxiliary α2δ‐1 subunit that is essential for of the L‐type Ca^2+^ channel activity (Bannister et al., 2009, 2012) was not changed in ANO1 knockdown mice, suggesting that this is an unlikely possibility.

ANO1 is known also to be important for Ca^2+^ homeostasis via interaction with IP_3_ receptors in sensory neurons (Jin et al., 2013) and the store‐operated Ca^2+^ channels (Forrest et al., 2010; Wang et al., 2016) in rat cerebral and pulmonary arteries. We did not find any functional support for changes of agonist‐induced Ca^2+^ release. However, an upregulation of guanine nucleotide‐binding protein subunit α11 in the ANO1 knockdowns, which mediates the pathway leading to phospholipase C activation and IP_3_‐dependent Ca^2+^ mobilization (Penn & Benovic, 2008), could be involved in Ca^2+^ response potentiation.

Proteomics identified also a significant upregulation of the Piezo1 channel in tail arteries downregulated for ANO1. The Piezo1 channel is Ca^2+^ permeable and mechanosensitive and is involved in smooth muscle contraction and arterial remodeling (Coste et al., 2010; Retailleau et al., 2015). We suggest that Piezo1 might be responsible for elevated Ca^2+^ influx during arterial contraction of ANO1 downregulated small arteries, although Piezo1 knockout was previously shown without effect on contraction induced by K^+^‐depolarization (Retailleau et al., 2015). The reason for this inconsistency with our data, where we have observed an increased K^+^‐induced contraction, is unclear. One might speculate that contraction‐associated increase in wall tension can stimulate the Piezo1 channels providing further Ca^2+^ raise and potentiation of contraction. This effect might be potentiated by increased Piezo1 expression, that is, in ANO1 downregulated tail arteries.

We also found that the sensitivity of the contractile machinery to intracellular Ca^2+^ was significantly decreased in all three ANO1 downregulated arteries in comparison with wild type arteries. Accordingly, we found an increased expression of Rho‐related GTP‐binding protein RhoE, which known to inhibit the Rho‐associated protein kinase (ROCK) and thus, suppresses Ca^2+^‐sensitization in the vascular wall (Loirand et al., 1999; Shimokawa et al., 2016). Thus, Ca^2+^ sensitization cannot explain the observed increase in contractility. This might be a compensatory mechanism reducing the effect of an increased Ca^2+^ influx, although this requires testing in further experiments.

Expression analysis further suggested phenotypical changes in ANO1 knockdown smooth muscle cells from tail arteries toward a contractile phenotype (Kudryavtseva et al., 2013). We found upregulation of a potent co‐activator of smooth muscle contractile genes, myocardin‐related transcription factor A (Liu et al., 2017; Velasquez et al., 2013; Zhang et al., 2007) in the tail arteries from ANO1 heterozygous mice. This increase might be associated with upregulation of Rho‐related GTP‐binding protein RhoB that is involved in hypoxia‐induced pulmonary artery contraction and remodeling (Wojciak‐Stothard et al., 2012) although its role in the peripheral circulation is not elucidated. The observed upregulation of plasma membrane Ca^2+^ ATPase (Gros et al., 2003) and troponin T (Kajioka et al., 2012) supports smooth muscle phenotypic switch toward a pro‐contractile phenotype of ANO1 knockdown smooth muscle cells.

The arterial function was assessed in this study using isometric myograph that does not entirely reproduce in vivo conditions (Falloon et al., 1995). A lack of some in vivo features, for example, myogenic activity, limits value of our experimental results for translation of ex vivo data to significance of blood pressure control. If saphenous arteries can be considered as a conductive artery, the tail artery in rodents is an important muscular artery that controls tail blood perfusion (Pang & Chan, 1985). With its role in thermoregulation, high myogenic activity, and dense innervation, it can be considered as a model for cutaneous resistance arteries in humans.

Since the role of ANO1 for contractility varied between different arteries and the arteries we studied here are unlikely to contribute substantially to the peripheral resistance, it was difficult to predict the consequences of ANO1 downregulation for blood pressure. Several previous studies indicated the importance of ANO1 for blood pressure regulation but the precise role is still controversial. Thus, ANO1 is strongly upregulated in pulmonary (Forrest et al., 2012; Sun et al., 2012) and primary (Wang et al., 2015) hypertension but reduced in secondary systemic hypertension (Wang et al., 2012). This suggests that these pro‐hypertensive changes in ANO1 expression can have a regional character affecting specific vascular beds. We did not find any changes in blood pressure in ANO1 heterozygous mice in comparison with wild type. This finding is somewhat surprising given that the previous finding of Heinze et al. (2014) where smooth muscle‐specific deletion of ANO1 led to reduced peripheral resistance. The reason for this inconsistency is unclear. These two genetically modified mice have different exons spliced out and are different on several other parameters though. In our mice, ANO1 protein expression is reduced, which is not the case in the other mice (Heinze et al., 2014). Moreover, our mice have ANO1 reduced globally in contrast to smooth muscle‐specific knockout in the previous study (Heinze et al., 2014). This enables the contribution of other neuronal and hormonal effects to smooth muscle development, phenotype, and function in vivo. Another important difference between these two genetically modified mice is that our mice are heterozygous while the mice used by Heinze et al. (2014) were homozygous. It is possible that partial ANO1 downregulation and, thus, only partial reduction of Cl^−^ conductance is insufficient to affect significantly the hemodynamic parameters. It might also be that the elevated contractility of the arteries, seen in our study, and reduced resistance by smaller arterioles, reported previously (Heinze et al., 2014) leaves total peripheral resistance at approximately normal level. This hypothesis needs to be tested experimentally in vivo. Moreover, our ANO1 heterozygous and wild type mice showed similar pressor and heart rate responses to phenylephrine injection and responses to massive vasorelaxation by Rho kinase inhibition. This suggests that the net‐effect of ANO1 downregulation in resistance vasculature is limited and that the baroreceptor function also appears to be intact.

## 5 CONCLUSION

In the present study, we describe a novel ANO1 heterozygous mouse model with deletion of exon 7 in one ANO1 allele. We show that smooth muscle cells from ANO1 heterozygous mice have reduced ANO1 expression and Ca^2+^‐activated Cl^−^ current when compared to wild type mice. This makes this mouse an important model to study the role of ANO1 in vascular function. Consistent with expectations, the contraction of aorta was reduced. Surprisingly, however, we observed increased contractility in tail and saphenous arteries from the heterozygous mice. We found that mechanistically this is likely due to elevated Ca^2+^ influx. Our data suggest that this could reflect smooth muscle phenotypic switch toward the pro‐contractile phenotype associated with upregulation of the Piezo1 channels in smooth muscle cells. This study challenges the conventional model for the role of ANO1 and suggests that ANO1 may have other functions than simply being responsible for a Cl^−^ conductance.

## CONFLICT OF INTEREST

None.

## AUTHOR CONTRIBUTION

BVS generated mouse model. HBJ, DK, AHJ, and HCB performed experiments. VVM, HBJ, DK, AHJ, and HCB made data analysis. VVM, HBJ, DK, AHJ, HCB, BVS, and CA took part in the conception, designed the study, and prepared the draft of the manuscript. VVM and CA completed the manuscript writing.

## Supporting information



Table S1

Table S2

Table S3
