# Supplementary material for: A paradoxical increase of force development in saphenous and tail arteries from heterozygous ANO1 knockout mice
Source: Physiol Rep. 2020 Nov 27;8(22):e14645. doi: 10.14814/phy2.14645 (PMC7695021; doi:10.14814/phy2.14645)
Supplement: Supplementary file 1 — Table S1 [file PHY2-8-e14645-s001.docx]

**Supplementary materials:**

**Table 1.** List of oligonucleotides used in the study.

| **Primer** | **Sequence** |
| --- | --- |
| TMEM_FAd1 | TGGTAAGCGGCCGCTTGTCCCAAGTCTAAACAGCACT |
| TMEM_FAr1 | TGGTAACGCGTAGGGGGTGTATGACATAATAGTTA |
| TMEM_FBd1 | TGGTAAGGATCCGGTCCTGCAAAGAGACCCTAAGC |
| TMEM_FBr1 | TGGTAAGTCGACGGGAACTGCTCTGGTTCTGACAC |
| TMEM_Ex7d1 | TGGTAACGCGTATAACTTCGTATAATGTATGCTATACG  AAGTTATGATATCCTACTGCACACAGCTGGCACCTTG |
| TMEM_Ex7r1 | TGGTAAGAATTCGTCCTTATATTCACACACACCCTCA |
| TMEM_S1d1 | TGGTAAGTCGACATCCTCTCCTCCCAGGGTCCAC |
| TMEM_S1r1 | TGGTAAGTCGACGAGTGGCCACACTATGGGATAAG |
| TMEM_ISD1 | TTGGCCTGTCTACTATTGATTA |
| TMEM_ISR1 | GAAATCTACCATGTGACCCAAC |

**Table 2** (Excel file). A summary of all proteins detected in tail arteries from ANO1 knockdown (Hz) and wild type (WT) mice and used in Ingenuity Pathway Analysis. Each of 5 WT probes as well as 3 Hz probes are from a single mouse, one Hz probe was a mixture of 3 mouse lysate because of limited amount of protein. Data compared by *t*-test.

**Table 3** (Excel file). Canonical pathways significantly affected by ANO1 knockdown and related to vascular metabolism and function as suggested by Ingenuity Pathway Analysis. P values, z-score and contributing proteins are listed.
